# Reciprocal Perspective for Improved Protein-Protein Interaction Prediction

**DOI:** 10.1038/s41598-018-30044-1

**Published:** 2018-08-03

**Authors:** Kevin Dick, James R. Green

**Affiliations:** Department of Systems and Computer Engineering, Carleton University, Ottawa, K1S 5B6 Canada

## Abstract

All protein-protein interaction (PPI) predictors require the determination of an operational decision threshold when differentiating positive PPIs from negatives. Historically, a single global threshold, typically optimized via cross-validation testing, is applied to all protein pairs. However, we here use data visualization techniques to show that no single decision threshold is suitable for all protein pairs, given the inherent diversity of protein interaction profiles. The recent development of high throughput PPI predictors has enabled the comprehensive scoring of all possible protein-protein pairs. This, in turn, has given rise to context, enabling us now to evaluate a PPI within the context of all possible predictions. Leveraging this context, we introduce a novel modeling framework called Reciprocal Perspective (RP), which estimates a localized threshold on a per-protein basis using several rank order metrics. By considering a putative PPI from the perspective of each of the proteins within the pair, RP rescores the predicted PPI and applies a cascaded Random Forest classifier leading to improvements in recall and precision. We here validate RP using two state-of-the-art PPI predictors, the Protein-protein Interaction Prediction Engine and the Scoring PRotein INTeractions methods, over five organisms: *Homo sapiens, Saccharomyces cerevisiae, Arabidopsis thaliana, Caenorhabditis elegans*, and *Mus musculus*. Results demonstrate the application of a *post hoc* RP rescoring layer significantly improves classification (p < 0.001) in all cases over all organisms and this new rescoring approach can apply to any PPI prediction method.

## Introduction

Elucidating the protein-protein interaction (PPI) network of an organism is necessary to understand cellular function and study disease pathogenesis^1^. Computational methods have been leveraged to rapidly estimate protein interaction networks. Given advances in compute power and more efficient prediction algorithms, we are now able to estimate comprehensive interactomes (*i.e*. all proteins in a proteome predicted against all others) for several species^2,3^. A number of paradigms have emerged in the field of PPI prediction including sequence-based^3,4^, structure-based^5^, evolution-based^6^, ontology-based^7^, and network-based methods^8^, each with varying degrees of success and compatibility^9^.

Historically, a broad range of machine learning algorithms have been applied to various facets of PPI prediction. Guo *et al*. demonstrated that Support Vector Machines (SVMs) used with auto-covariance can be successfully applied to sequence-based PPI prediction tasks^10^. Hamp and Rost expanded upon this work with the incorporation of evolutionary profiles to generate profile-kernel SVMs, demonstrating further improvements to PPI classification performance^11^. Neural networks with varying model architectures have also been successfully leveraged and most recently, Sun *et al*. have demonstrated that stacked denoising autoencoders (an artificial neural network which infers a function to construct hidden structures from unlabeled data) can achieve similar successes to predict PPIs^12^. The sites of a protein which mediate PPIs have also been predicted algorithmically^13^ and using Random Forest ensemble models leveraging features extracted from the physiochemical properties of protein structures^14^. More recently, Wang *et al*. used a Rotation Forest ensemble approach leveraging multiple sequence alignments which was found to be successful in the large-scale identification of PPIs in several species^15^. Overall, the field of PPI prediction has been highly active in the last decade, with new methods proposed each year as recently reviewed in Kotlyar *et al*.^16^. While the field of PPI prediction is methodologically diverse, irrespective of the paradigm, learning algorithm, and scale of the number of predictions, the field has certain fundamental commonalities.

All of these methods examine the query protein pair and output a score denoting the predicted likelihood that the pair will physically interact. Despite the methodological differences between each paradigm, common to each is the intrinsic need to apply a decision threshold to the score that distinguishes the set of high-confidence predicted PPIs, potentially warranting experimental validation, from the set of protein pairs that are unlikely to physically interact. Establishing the expected number of PPIs for any given protein is an openly debated question, which is further complicated given that a protein can exhibit context-specific binding patterns^17^. While some proteins can exhibit promiscuous binding behaviours, as in the case of hub proteins, others are highly specific with few binding partners^18^. The selection of an appropriate decision threshold remains an open question and directly influences the performance of a PPI predictor.

## High Throughput Predictions Give Rise to Context

Historically, PPI prediction tasks have been limited to modest subsets of the complete interactome enabling the elucidation of localized sub-networks or the identification of an interspersion of isolated PPIs relative to the complete interactome. This limitation is due to the algorithmic time-complexity of most PPI predictors, particularly those examining protein structure. Given that the number of putative PPIs grows as the square of number of proteins (*i.e*. the triangular number), the computational time for a given PPI using a given predictor is critical. For example, the human proteome contains over 20,000 proteins representing over 200 million potential interactions. A method predicting one interaction per second would require over 6.3 years to examine all pairs and produce the complete human interactome. This has prompted research groups to develop optimised predictors and leverage high performance computing. Only recently have these high-throughput predictors afforded us the capability to scale predictions and consider the comprehensive set of all possible interactions^2,3^. This has given rise to context: the ability to appraise a given PPI prediction relative to all possible interactions. While high-throughput methods have now enabled the study of a given PPI within the context of all protein pairs, the interpretation of these results is critical to appropriately evaluate the PPI predictor.

Traditionally, cross-validation experiments are used to evaluate the performance of a trained predictor: a random subsample of all interactions are retained, the remaining data are used to train a model, and the subsample is then evaluated. Receiver Operating Characteristic (ROC) curves are widely used to compare the performance between models and are leveraged to select an operating threshold that balances sensitivity and specificity. The area under the ROC curve (AUC) is often used to summarize a model’s performance across all possible decision thresholds, although the value of such a summary statistic is questionable, particularly in the case of highly imbalanced problems such as PPI prediction. Increasingly, the suitability of ROC curves and the AUC metric is being reconsidered in various fields^19–21^, and prevalence-corrected Precision-Recall Curves (PRCs) have been adopted as the new standard^19^.

Once a decision threshold is selected, it is applied globally to all predictions and is used as a binary discriminator: predicted PPIs scoring above the threshold are considered positives, warranting further investigation, whereas those below are considered negatives. Tuning this threshold to less conservative levels threatens to introduce a large number of false positives, thereby reducing the utility of the classifier.

## Related Work and Previous Approaches to Local Threshold Determination

In PPI prediction tasks, a quantitative score can be assigned to a given protein pair of interest, say proteins *x*_*i*_ and *y*_*j*_ in pair *P*_*ij*_. Researchers studying protein *x*_*i*_ would typically consider the set of sorted scores for all pairs ${P}_{in},\forall \,n$ and investigate the top-ranking PPIs through experimental validation. However, the arbitrary selection of the top-k ranking interactors for a given protein fails to impart any confidence in the resulting PPIs. The choice of the value of *k* is arbitrary since no single value of k can be optimal for all proteins. Furthermore, when considering the interaction *P*_*ij*_, this top-*k* ranking approach only considers the scores of pairs involving *x*_*i*_, but not all pairs involving *y*_*j*_. That is, they are only leveraging half of the available context.

A widely used algorithm that does examine both partners within a putative relationship is the Reciprocal Best Hit method to identify putative orthologs. Here, two genes in different genomes are considered to be orthologs if and only if they find each other to be the top-scoring BLAST hit in the other genome^22^. Reciprocal Best Hit is an example of the most conservative application of a local threshold, where *k* = 1. While useful for determining an orthologous relationship between the two genes that are typically expected to have a single ortholog in other species, our situation differs as proteins are expected to participate in several PPIs (*i.e*. $k\ge 1$); therefore, a more suitable approach is required.

A similar challenge arises in the control of false discovery rates (FDRs) in applications such as genomics where high dimensional genotyping arrays are used to evaluate millions of variants (*e.g*. single nucleotide polymorphisms) for correlation with phenotype or experimental condition. The established method for controlling against multiple testing has been to adjust family-wise error rates (FWERs) such as using the Holm-Bonferroni method^23^. The control of FWERs has been considered too conservative and severely compromises statistical power resulting in many true loci of small effect being missed^24^. Recently local FDRs (LFDRs) have been proposed and are defined as the probability of a test result being false given the exact value of the test statistic^25^. The LFDR correction, through re-ranking of test statistic value, has been demonstrated to eliminate biases of the former non-local FDR (NFDR) estimators^24,26^. Our application is similarly motivated, though differs in that we consider the paired relationships between two elements and leverage the context of a given protein relative to all others.

The network-based analysis of PPI networks contextualizes proteins using undirected connected graphs, often with a scale-free topology and hierarchal modularity^27^. The importance of a PPI is determined by considering topological features, path distances, and centrality measures of a given protein relative to the neighbours in its vicinity (*e.g.* *k*-hop distance). While useful for *post hoc* evaluation of cluster density, cliques, and protein complex prediction^28^, these approaches are notoriously plagued with false positives^29^. Network-based PPI prediction methods will attribute scores based on these quantitative metrics and often incorporate external information to re-weight their score such as protein localization, co-expression, and literature-curated data.

In essence, various methods have taken into account localized decision thresholds, paired comparison, PPI context, and rank-order metrics, however no one modality has leveraged these in combination and proposed a unified method to determine localized decision thresholds for predicted PPIs based on their interactome context. Again, such analysis has only recently become possible with the development of efficient high-throughput methods capable of assessing all possible protein pairs.

## The Challenge with Selecting Decision Thresholds

A central problem to PPI prediction is the wide variance observed in the number of actual interactions of each protein (the degree from network-based terminology). This is further complicated when considering protein binding site characteristics, where some proteins exhibit highly specific interaction profiles, while others have a tendency towards promiscuity due to large (and at times, overlapping) binding sites^30,31^ wherein the same polypeptide will participate in several interactions. Furthermore, the subset of proteins known to exhibit promiscuity, given their high affinity binding sites, tend to have an increased probability to develop new connections over time relative to proteins exhibiting modest binding affinities^32^. These hub proteins have the tendency to interact with considerably larger number of proteins and often have physiochemical sites (structured or unstructured) enriched with intrinsic disorder enabling an above-average propensity to interact with other proteins^33–35^.

PPI predictors trained with known interactions involving promiscuous proteins risk developing a bias towards these windows, thereby producing an inflated predicted score for such proteins relative to others. While useful for the identification of hub proteins, resulting PPI models risk having an over-representation of these windows and an under-representation of their highly specific counterparts. Given that true PPIs are rare and PPI predictors tend to over-predict interactions for some proteins while under-predicting for others, we aim to quantify this behaviour and incorporate it into the decision function. True interactions are rare among all possible pairs and predictors exhibiting a bias towards certain proteins is problematic. For example, should a PPI predictor, when applied to a given protein *A*, produce a large number of high-scoring targets, one naturally questions the validity of each score and would favor a conservative decision threshold, *D*_*A*_. Conversely, when applied to protein *B* and only a small number of high-scoring targets are observed, even if these few are low relative to the stringent decision threshold *D*_*A*_, one might be inclined to pursue these few predictions further.

**Figure 1:**
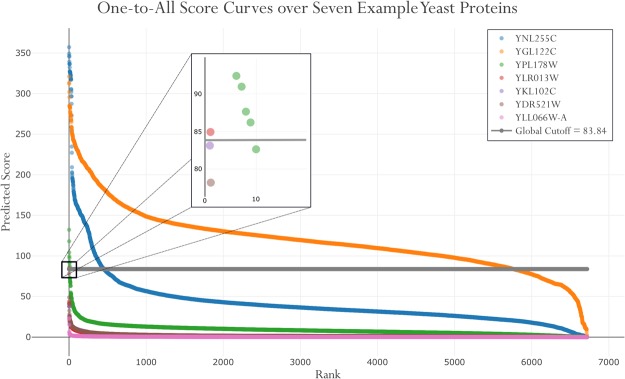
One-to-All Score Curve over Seven Example Yeast Proteins. The rank order distribution of all predicted scores with a given protein (*i.e*. one-to-all) are compared against the selected decision threshold (Global Cutoff), depicted as the grey line (score = 83.84) and determined by leave-one-out cross-validation at a specificity of 99.95%. Despite being extremely conservative, this threshold is clearly inappropriate for certain example proteins, namely YGL122C (the Nuclear polyadenylated RNA-binding protein, NAB2) as ~5,800 of the 6,717 putative protein interactions are considered positives. Conversely, YLR013W, YKL102C, YDR521W, and YLL066W-A are all predicted to have no true interactions at this threshold. Only YNL255C and YPL178W appear well matched to the global threshold with ~400 and 9 positively predicted interactions respectively, reflecting the diversity in the number of true interactions.

Figure 1 illustrates this phenomenon with a *One-to-All Score Curve* using seven example proteins from the *Saccharomyces cerevisiae* predicted interactome^36^, where PIPE was used to predict all scores between the yeast proteome and seven diverse yeast proteins. These One-to-All Score Curves depict the rank order distribution (*i.e*. the sorted descending ordering of scores) of a single protein. When plotted together, these curves illustrate the comprehensive set of predicted scores for one protein, enabling researchers to interpret each distribution relative to other proteins and compared to the globally defined decision threshold (Fig. 1, in grey). An obvious question arises for a protein such as the UPF0479 Membrane Protein (in pink): if no PPIs score above the defined global threshold, how might we determine the number of interactions to consider? Tuning the global threshold to a lower point threatens to introduce an exorbitant number of lower-confidence PPIs with a protein such as the Nuclear polyadenylated RNA-binding protein (in orange). Here we propose a method to define a local threshold on a per-protein basis which addresses these challenges and more appropriately defines what constitutes a positively and negatively predicted PPI. Beyond the definition of a localized per-protein threshold, a PPI can be further contextualized through paired comparison.

## Reciprocal Perspective

We introduce a novel concept in the application of interactome-wide analyses of protein-protein interaction networks: Reciprocal Perspective (RP), which jointly considers an interaction from the perspective of each partner to determine a new assessment of the interaction. Intuitively, when two elements interact, that interaction may have differing implications from each element’s respective perspective. Therefore, the relationship can be characterized from the two perspectives separately, taking into account the context relevant to each element. We define *perspective* as the set of relationships of one element with all others. Applied to PPI predictions, the “elements” correspond to proteins and the “relationships” are PPIs. Simply put, when considering a putative interaction between elements A and B (A-B), one can examine A-B in the context of all of A’s putative interactions (A-*) and also in the context of all of B’s putative interactions (*-B). Here, we formalize these concepts into a generalized method termed RP-PPI (Reciprocal Perspective for PPI prediction) and demonstrate its ability to improve the classification accuracy of two state-of-the-art PPI predictors using a cascaded Random Forest classifier.

## Methods

Inspired by the visualization of the rank order distribution of PPIs for selected proteins (Fig. 1), we seek to develop a method to determine the optimal local threshold for each protein. Noting that, while the average magnitude of each curve varies, these distributions share a characteristic S-curve shape, with a high scoring region, a baseline region, and a low scoring region (the latter is sometimes missing, thereby forming an L-curve shape). We first seek to determine a robust procedure to estimate this baseline, with the ultimate goal of differentiating the high scoring region. To examine a predicted PPI in the context of the baseline level, a number of PPI re-scoring metrics are introduced as functions of the estimated baseline. Finally, reciprocal perspective is applied to develop a number of reciprocal rank order metrics to characterise a given PPI. These new PPI re-ranking metrics are validated using two state-of-the-art PPI predictors over five organisms: *Homo sapiens*, *Saccharomyces cerevisiae*, *Arabidopsis thaliana*, *Caenorhabditis elegans*, and *Mus musculus*.

### Modeling the Curve

The generation of the set of comprehensive PPI predictions using the two state-of-the-art methods for each of the five organisms is described in the Evaluation Design section and their results were used here. An examination of several one-to-all PPI score curves reveals a characteristic “S”- or “L”-shaped curve with a certain number of higher-scoring PPIs relative to a large baseline. Due to the inherent class imbalance in PPI prediction tasks, true interactions are rare. We therefore assume that the baseline region corresponds to the typical score assigned to a non-interacting protein pair involving the query protein. The baseline level thus provides a proxy for estimating the degree of bias that the predictor has for the query protein; as described above, some proteins will universally produce higher prediction scores than others. We therefore seek to estimate this baseline level and identify those putative interactions residing significantly above this baseline score.

**Figure 2:**
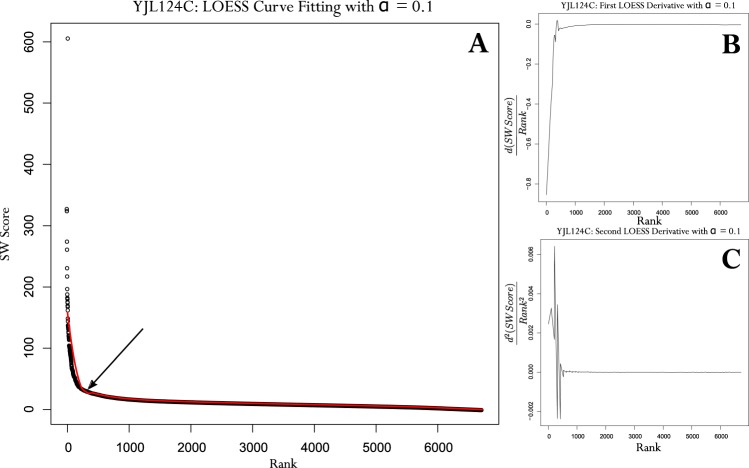
One-to-All Score Curve of YJL124C with fitted LOESS curve and corresponding first (**B**) and second derivative (**C**) curves. Arrow indicates the knee of the curve.

To differentiate between high-scoring PPI and baseline, we seek to identify the knee of these curves. A continuous smooth curve is therefore fit to the one-to-all score curves and the maximum value of the second derivative of the fitted curve represents the knee. Given the variability of the shapes of the curves we do not assume a specific distribution and use the non-parametric Locally Weighted Regression (LOESS) method to fit low-order polynomials (degree = 2) to local windows of scores to produce a piecewise continuous curve^37^. LOESS curve fitting is parameterized by the *span* parameter, *α*, which determines the proportion of points considered in a window and controls the degree of smoothing. Parameter tuning was accomplished by varying *α* over the [0.05, 0.75] range and observing the quality of curve fit over a stratified sample of 1% of proteins. The space of one-to-all score curves was stratified and visualized by first sorting all curves by their maximum score and then plotting a surface over all curves in a third dimension in increments of 100 proteins. We could therefore qualitatively appraise the fit of the curve with a given *α* over the space of all one-to-all curve shapes by randomly selecting a representative from each increment, accounting for 1% of the complete dataset (repeated for each method and organism dataset). A preliminary coarse-grained analysis restricted our range to the [0.05, 0.20] range, wherein a fine-grained analysis varying *α* by 0.01 was used to select the final value. Repeating the stratified sampling procedure, a value of *α* = 0.10 was selected as it provided qualitatively the best fit and generalized across both methods and for distributions across the five organism datasets. Fig. 2 illustrates an example of the LOESS curve fit to the one-to-all scores for protein YJL124C and the corresponding first and second derivative curves based on the original fit. The piecewise linear characteristic of Fig. 2A depicts the rapid rate of decline of the rank order values followed by the gradual decline beyond the knee of the curve. The maximum value in the second derivative curve (Fig. 2C) corresponds to the point of greatest upwards concavity in the original plot; we establish this as the knee and the starting point of the baseline (Fig. 2A; arrow). Having established this knee for each protein we wish to restate each putative interactor’s score with respect to this baseline. To that end, we defined several quantitative metrics.

### Notation

We define our metrics using set theory. We first define the set of proteins for a given organism *a* as $A=\{{x}_{1},{x}_{2},\ldots ,{x}_{n}\}$. Organism *a* is said to have a proteome of size *n*. Similarly, organism *b* has a set of proteins defined as $B=\{{y}_{1},{y}_{2},\ldots ,{y}_{m}\}$ and a proteome of size *m*. We define ${x}_{i}{y}_{j}\mathrm{\ |\ }{x}_{i}\in A,{y}_{j}\in B,i\in \mathrm{\{1,}\,\mathrm{2,}\ldots ,n\},j\in \mathrm{\{1,}\,\mathrm{2,}\ldots ,m\}$ as a predicted binary PPI with symmetric relation: $\forall \,x,y\in C(xRy\iff yRx)$ where *C* is the set of all possible interactions between organisms *a* and *b*:

1 $$C=\{{x}_{1}{y}_{1},{x}_{1}{y}_{2},\ldots ,{x}_{i}{y}_{j},\ldots ,{x}_{n}{y}_{m-1},{x}_{n}{y}_{m}\}$$

and *R* represents the binary protein-protein interactions relation over the set *C*.

In the case where we consider intra-species interactions (*i.e*. organism *a* is also organism *b*), we satisfy $\forall \,z[z\in A\leftrightarrow z\in B]$. Furthermore, we define the score and rank order for a given interaction, *x*_*i*_*y*_*j*_. The score of a given PPI, ${x}_{i}{y}_{j}\sim {y}_{j}{x}_{i}$, is defined as ${s}_{{x}_{i}{y}_{j}}={s}_{{y}_{j}{x}_{i}}$ and these values are used to sort the respective sets. The ordered set of interactions involving protein *x*_*i*_ is defined as the totally ordered set $X=\{{x}_{i}{y}_{k},\ldots ,{x}_{i}{y}_{l}\mathrm{\}\ |\ }k,l\in \mathrm{\{1,}\,\mathrm{2,}\ldots ,m\}$ and the set involving protein *y*_*j*_ is similarly defined as $Y=\{{y}_{j}{x}_{u},\ldots ,{y}_{j}{x}_{v}\mathrm{\}\ |\ }u,v\in \mathrm{\{1,}\,\mathrm{2,}\ldots ,n\}$. We, therefore, define the rank order for *x*_*i*_*y*_*j*_ as ${r}_{{x}_{i}{y}_{j}}$, the ordinal rank of *x*_*i*_*y*_*j*_ in *X*, and ${r}_{{y}_{j}{x}_{i}}$ as the ordinal rank of *y*_*j*_*x*_*i*_ in *Y* and say that ${r}_{{x}_{i}{y}_{j}}$ and ${r}_{{y}_{j}{x}_{i}}$ are reciprocal rank order (RRO) values.

Taking into account the knee of the curve as the baseline threshold obtained from the second derivative of the continuous curve using LOESS, denoted *τ*, we obtain useful definitions specific to each protein, namely ${s}_{{x}_{\tau }}$ as the score corresponding to $max(\frac{{d}^{2}({s}_{x})}{{x}^{2}})$ and ${r}_{{x}_{\tau }}$ as its ordinal rank value. In the partner protein, we similarly define ${s}_{{y}_{\tau }}$ and ${r}_{{y}_{\tau }}$. A predicted PPI can then be compared relative to these baselines in a number of ways, resulting in various metrics to define the score of a putative PPI relative to the baseline level. Here, we use a binary value ${\beta }_{{x}_{i}{y}_{j}}\in \mathrm{\{0,}\,\mathrm{1\}}$ to denote whether ${s}_{{x}_{i}{y}_{j}}$ resides above or below ${s}_{{x}_{\tau }}$, the binary variable ${\beta }_{{y}_{j}{x}_{i}}\in \mathrm{\{0,}\,\mathrm{1\}}$ to denote whether ${s}_{{y}_{j}{x}_{i}}$ resides above or below ${s}_{{y}_{\tau }}$, and a binary variable ${\gamma }_{{x}_{i}{y}_{j}}\sim {\gamma }_{{y}_{j}{x}_{i}}\in \mathrm{\{0,}\,\mathrm{1\}}$ to denote whether ${s}_{{x}_{i}{y}_{j}}={s}_{{y}_{j}{x}_{i}}$ resides above or below the global threshold. Furthermore, the respective fold-differences from the baseline are defined:

2 $$F{D}_{{x}_{i}{y}_{j}}=\frac{{s}_{{x}_{i}{y}_{j}}-{s}_{{x}_{\tau }}}{{s}_{{x}_{\tau }}}$$

3 $$F{D}_{{y}_{j}{x}_{i}}=\frac{{s}_{{y}_{j}{x}_{i}}-{s}_{{y}_{\tau }}}{{s}_{{y}_{\tau }}}$$

From the estimated baseline of a given protein’s perspective, we obtain the following five metrics, which we summarize in Table 1: Rank-XY, Rank-Local-Cutoff-X, Score-Local-Cutoff-X, Interaction-XY-Above-Local-X, Fold-Difference-From-Local-X. Considering that each putative PPI has two perspectives, we obtain an additional five metrics from the reciprocal perspective: Rank-YX, Rank-Local-Cutoff-Y, Score-Local-Cutoff-Y, Interaction-YX-Above-Local-Y, Fold-Difference-From-Local-Y. Furthermore, these can be utilized to derive metrics characterizing each PPI and jointly accounting for the context of the putative PPI within each protein’s respective perspective.

**Table 1:** Reciprocal Perspective Features for Protein-Protein Interaction Prediction.

Feature Name	Notation	Feature Type	Description
Rank-XY	${r}_{{x}_{i}{y}_{j}}$	Rank	The rank order of Protein Y among all of the predictions for Protein X
Rank-YX	${r}_{{y}_{j}{x}_{i}}$	Rank	The rank order of Protein X among all of the predictions for Protein Y
Naïve Rank Order	*NaRRO*	Rank	As defined in Reciprocal Perspective Notation
Normalized Rank Order (Proteome X)	*NoRRO*_*A*_	Rank	As defined in Reciprocal Perspective Notation
Normalized Rank Order (Proteome Y)	*NoRRO*_*B*_	Rank	As defined in Reciprocal Perspective Notation
Adjusted Rank Order	*ARRO*	Rank	As defined in Reciprocal Perspective Notation
Rank-Local-Cutoff-X	${r}_{{x}_{\tau }}$	Rank	Rank order of the protein nearest to the local cutoff value of Protein X
Score-Local-Cutoff-X	${s}_{{x}_{\tau }}$	Score	Score at the local cutoff value of Protein X
Rank-Local-Cutoff-Y	${r}_{{y}_{\tau }}$	Rank	Rank order of the protein nearest to the local cutoff value of Protein Y
Score-Local-Cutoff-Y	${s}_{{y}_{\tau }}$	Score	Score at the local cutoff value of Protein Y
Interaction-XY-Above-Local-X	${\beta }_{{x}_{i}{y}_{j}}$	Rank	Binary variable indicating whether the interaction XY is above the local cutoff of protein X
Interaction-YX-Above-Local-Y	${\beta }_{{y}_{j}{x}_{i}}$	Rank	Binary variable indicating whether the interaction YX is above the local cutoff of protein Y
Above-Global-Threshold	${\gamma }_{{x}_{i}{y}_{j}}\sim {\gamma }_{{y}_{j}{x}_{i}}$	Score	Binary variable indicating whether the Original Score is greater than the globally determined cutoff value
Fold-Difference-From-Local-X	$F{D}_{{x}_{i}{y}_{j}}$	Fold	As defined in Reciprocal Perspective Notation
Fold-Difference-From-Local-Y	$F{D}_{{y}_{j}{x}_{i}}$	Fold	As defined in Reciprocal Perspective Notation

A simplified feature name is defined; the notation is kept consistent with that of section Reciprocal Perspective Notation.

### Reciprocal Perspective Notation

For a given protein within a pair, a set of metrics can be determined to rescore the predicted PPI relative to all other PPIs involving that protein, taking into account its estimated baseline. However, this examines the PPI from only a single perspective. RP examines a putative PPI in the context of both proteins in the pair. By jointly considering these reciprocal perspectives, RP may be applied as a post-processing step to the raw predicted scores to augment the predicted outcomes by supplying additional context to previously generated scores and produce the final output classification. Having defined new metrics in the context of a protein’s perspective, we now define those which combine information from each perspective. In its simplest form, the Naïve Reciprocal Rank Order (NaRRO) value is the product of the rank order values, inversed to cast into the $[\frac{1}{nm},\,\mathrm{1]}$ range.

4 $$NaRRO={({r}_{{x}_{i}{y}_{j}}{r}_{{y}_{j}{x}_{i}})}^{-1}$$

This representation doesn’t account for the proteome size and the potentially imbalanced number of predictions when comparing predicted values from different species. To account for inter-species predictions where each organism has a differently sized proteome (such as predicting PPIs between host and pathogen), we must normalize the NaRRO value to avoid artificially inflated values from smaller proteomes. To correctly normalize scores for inter-species interactions we define the Adjusted Reciprocal Rank Order (ARRO); we normalize each rank order value with the respective proteome size:

5 $$ARRO={(\frac{{r}_{{x}_{i}{y}_{j}}}{n}\times \frac{{r}_{{y}_{j}{x}_{i}}}{m})}^{-1}$$

This corrects any biases due to differently sized dataset in inter-species predictions. ARRO values are positive float values in the range $\mathrm{[1,}\,nm]$.

Finally, for the case of intra-species predictions (*i.e. A* = *B*), we simplify the ARRO notation to define the Normalized Reciprocal Rank Order (NoRRO) which normalizes each RRO value by dividing by the proteome size, where *p* is the proteome size, *p* = *n* = *m*.

6 $$NoRRO={(\frac{{r}_{{x}_{i}{y}_{j}}{r}_{{y}_{j}{x}_{i}}}{p})}^{-1}$$

This metric is generally appropriate only for intra-species predictions and is useful to compare scores across different species. The NoRRO values are positive float values in the range $[\frac{p}{nm},p]$. A special case arises when considering the NoRRO metric in the context of inter-species predictions, where organisms with differently sized proteomes would require the selection of either as normalization factor. Exploration of this use case is deferred to future study and beyond the scope of this current work. These 15 new metrics (10 protein-specific; five derived from RP) are summarized in Table 1 and are each amenable to the analysis of the PPI prediction results of any PPI predictor over any organism, or combination there-of.

### Evaluation Design

We validated our method using the comprehensive set of predicted scores from two state-of-the-art PPI prediction methods and on five organisms: *H. sapiens*, *S. cerevisiae*, *A. thaliana*, *C. elegans*, and *M. musculus*. The PPI predictions methods were selected since they are uniquely amenable to the comprehensive prediction of interactomes: the Protein-protein Interaction Prediction Engine (PIPE)^2,4,13^ and the Scoring PRotein INTeractions (SPRINT)^3^ algorithms. PIPE has successfully been used in the comprehensive prediction of a number of intra- and inter-species interactomes since its release in 2006 including *H. sapiens*, *S. cerevisiae*, Human Immunodeficiency Virus, the Zika Virus, *Plasmodium falciparum*, and *Glycine max*^2,4,38,39^. Leveraging pairs of known interacting proteins, the PIPE algorithm identifies subsequence regions of similarity (termed “windows”) and uses these windows to determine whether or not pairs of unknown PPIs are likely to interact. High frequency windows present challenges as they may artificially inflate predicted scores for those proteins that contain them. Efforts to normalize variable frequency windows have previously been proposed, most notably with the introduction of the Similarity-Weighted (SW) scoring metric used in PIPE3, which normalizes the PIPE-Score for a given window by the frequency of that window throughout the training dataset. The SW score is a positive, float value which captures the likelihood of interaction between any two proteins. We consider this as the original score in the RP method.

The SPRINT algorithm is also a sequence-based PPI predictor amenable to the comprehensive prediction of all possible PPIs. It distinguishes itself from similar predictors by using a multiple spaced-seed encoding to prune away high frequency elements that occur too often to be involved in PPIs, and then returns a score indicative of the probability to interaction^3^. It has been compared to several state-of-the-art methods including PIPE2, as in^40^. For *H. sapiens*, the SPRINT method has been postulated to out-perform the PIPE2 algorithm, suggesting it is comparable with the state-of-the-art^3^. This work used the PIPE3 (*i.e*. MP-PIPE) model which was previously successful in generating the first comprehensive *H*. *sapiens* interactome^2^. An explicit comparison of the SPRINT and PIPE3 methods is beyond the scope of this work and here the two are used as independent methods capable of generating predicted scores for any given PPI, and therefore are amenable to analysis using RP.

PPI predictions for five different organisms were used to validate the RP method. To generate the comprehensive set of interactions, the high quality PPI datasets to train and evaluate each method were obtained using the Positome framework^41^. Specifically, the “conservative” dataset, as defined in^41^ was acquired for each organism except for the *C. elegans* where the “permissive” dataset was used following the recommendations of^41^ for organisms where the conservative dataset is excessively stringent resulting in very few training samples. This framework pre-processes the data and ensures a consistent definition of a PPI and provides the corresponding protein sequences data for all proteins present in the dataset. The training data for each of the five organisms is summarized in Table 2. The comprehensive prediction (*i.e*. “all-to-all”) was obtained using the respective default parameters for each of the PIPE and SPRINT prediction methods for each organism.

**Table 2:** Summary of Training Data for the Five Organisms.

Organism	Number of Proteins	Number of Training PPIs
*H. sapiens*	20,160	13,938
*S. cerevisiae*	6,717	74,608
*A. thaliana*	16,886	3,027
*C. elegans*	6,443	7,923
*M. musculus*	17,759	2,938

With the all-to-all datasets computed for each organism over each method, the global threshold was determined for each model and for each organism. The leave-one-out cross-validation (LOOCV) method will remove a known interaction from the positive training dataset, build a model on the remaining, and then predict that removed interaction. It is considered the computational equivalent of wet-lab experimental validation of a PPI and useful in estimating the global decision threshold of a given predictor. Given the large class imbalance, it is crucial to select a decision threshold which minimizes the number of false positives. For both methods, the specificity was set as 99.95% and the corresponding decision threshold was chosen as in^2,42^.

The RP method was then used to compute the defined metrics for each PPI and a feature matrix was generated. To evaluate the improvement provided by the RP post-processing method, the set of positive PPIs and an equivalently sized subsample of negative PPIs (sampled without replacement) was created to train and evaluate a Random Forest classification model. We used the *scikit-learn* Python library default parameters where tree depth was unbounded and feature selection occurred over the root of the number of features. We selected a forest size of *t* = 100 trees in favor of the default *t* = 10 since a finer-grained classification confidence and numerical precision could be obtained from the *t* number of trees. We opt to arbitrarily select these hyperparameters as we cannot expect that there exists a singular set of Random Forest hyperparameters which will uniquely lead to the best increase in performance over all methods and all organisms. Moreover, using the same set of hyperparameters in our Random Forest models enables us to appropriately compare the results between the two principal conditions (RP-Enhanced vs. Original) for a single dataset, as well as observe consistent improvement in predictive performance across all methods and organisms. The feature space with respect to *t* and PRC-AUC was explored and summarized in Supplementary Figure S1, available online.

We selected this machine learning method given that they have been demonstrated to be robust against overfitting, relatively simple to tune, and generally outperform standard classifiers^43^. Each model was produced using a five-fold cross-validation to further avoid overfitting the data. The feature subsets types (“Rank”, “Score”, “Fold”) of the post-processed RP features, from Table 1 were tested in combination to delineate their contribution to the discrimination of the positive and negative class. We refrained from further tuning the hyper-parameters so as to consistently evaluate each test condition. These results were plotted on prevalence-corrected precision-recall curves. Since the class imbalance in our test data is not necessarily reflective of the actual degree of imbalance expected when the classifier is applied to a complete genome, we use the prevalence-corrected precision defined as:

7 $$Sn=recall=\frac{TP}{TP+FN}$$

8 $$Sp=\frac{TN}{TN+FP}$$

9 $$precision=\frac{TP}{TP+FP}=\frac{Sn}{Sn+r\mathrm{(1}-Sp)}$$

where *TP* is the estimated true positive rate of the classifier, *FP* is the estimated false positive rate of the classifier, *r* is the expected ratio of negative to positive samples in the real-world data, *Sn* is the estimated sensitivity, and *Sp* is the estimated specificity of the classifier. The precision is interpreted as the portion of positively predicted PPIs that are actually true interactions whereas the recall is interpreted as the proportion of positives that were correctly classified as true. Here we use a value of 100 for *r* representing a 1:100 class imbalance, as estimated and used in^40,42^. Finally, to quantify the statistical difference between the non-RP and RP-enhanced conditions we used bootstrap testing over 1,000 iterations and summarize the resulting PRC and ROC curves using the area under the curve metrics: PRC-AUC, ROC-AUC.

### Data availability

The data that support the findings of this study are available from BioGrid (known PPIs) and UniProt (protein sequences).

## Results and Discussion

Throughout the history of PPI prediction, and in general applications of machine learning, the question of selecting an appropriate decision threshold *τ* has been a source of much debate. Applications with balanced and equivalently valued classes can simply select the point which jointly maximizes the sensitivity and specificity of an ROC curve (*i.e*. the threshold ${\tau }^{\ast }$ nearest to [0, 1] of the ROC curve). As the class imbalance varies and the relative cost of a *FP* versus a *FN* changes, the selection of a decision threshold becomes increasingly critical. The arbitrary selection of τ^*^ threatens to flood the prediction results with Type I errors (assuming a class imbalance *pos*:*neg* where $pos\ll neg$). To account for this, the trade-off between sensitivity and specificity can be adjusted such that the specificity is valued much more (*i.e*. reduction of the false positive rate) at the expense of the true positive rate. As a result, we have an increased confidence in the predicted positives (high precision), however this comes at the cost of missing a majority proportion of actual positives (high number of Type II errors).

This conservative approach has been demonstrated to be successful in the elucidation of novel PPIs when predicting across the entire interactome of various organisms^2,4,42^; however, the visualization of predicted scores relative to that globally applied decision threshold provides insight into the distribution of the scores of a given PPI in the context of each participating protein relative to all others within the proteome. Most notably, we observe that the definition of a single conservative and globally applied decision threshold appears to be appropriate for a subset of proteins and inappropriate for others (Fig. 1). These one-to-all curves characterize the distribution of predictions for a given protein against all others within the proteome. Having a totally rank-ordered distribution, one can immediately appraise a number of important properties such as the values, size, and range of both the highest scoring PPIs and the baseline, as well as the location of the knee of the curve. Investigating the distribution of scores across all interacting proteins, we observe some proteins with a disproportionately large number of retained interactions (where putative PPI scores are above the global threshold). These might occur for two reasons: biologically, the protein windows have a high binding affinity, or computationally, the PPI predictor overestimates the predictions. The converse is also likely, wherein certain proteins exhibit having very few, or no predicted PPIs score above the global decision threshold when interpreting the One-to-All curve. Realizing that a large number of predicted PPIs on this One-to-All curve score highly relative to the other predicted PPIs despite falling below the global decision threshold prompted the formulation of hypotheses that those putative PPIs might comprise a proportion of false negatives. This opens the opportunity to appraise a given protein in the context of the interactome, something that has only recently become possible with the ability to generate comprehensive interactomes.

Generating a score for all possible protein pairs is a computationally demanding process. Historically, only targeted subsets of protein pairs were considered and therefore did not face scalability challenges. The size of the comprehensive set of all intra-species predictions is the triangular number of the size of the proteome, $\frac{n(n+\mathrm{1)}}{2}$; the number of required predictions grows in *O*(*n*^2^) necessitating predictors capable of producing predictions on the order of the fraction of a second. With the advent of such predictors and the availability of cloud computing resources, such interactomes are available and give rise to context: the ability to appraise a given interaction in relation to all others. This enables the opportunity to estimate the baseline on a per-protein basis and derive quantitative metrics of a given PPI in relation to all others and inspired the development of the RP framework.

### Global to Local: A Single Global Decision Threshold is Inappropriate

Leveraging visualization techniques, we observe considerable variation in the distribution of comprehensive predictions between the one-to-all curves of various proteins (Fig. 1). This is not unexpected, given the variation of binding affinities exhibited by different proteins. However, relative to the globally defined decision threshold delineating those PPIs considered as positively interacting from the negatives, we observe the simultaneous over- and under-representation of different proteins. Whereas proteins with high affinity windows will have the tendency to “pull up” the decision threshold, proteins with highly specific windows will be excluded from the set of positively interacting PPIs. We note that these proteins exhibit a rare few PPIs scoring higher than the others on the One-to-All curve, providing evidence for their putative interaction despite falling below the global decision threshold. To address this, the definition of a localized threshold on a per-protein basis is required. Furthermore, generating the comprehensive set of predictions guarantees that every protein can be consistently evaluated in relation to all compliments and vice versa, motivating the development of methods leveraging these reciprocal perspectives.

### Reciprocal Perspective Significantly Improves Classification Accuracy

**Figure 3:**
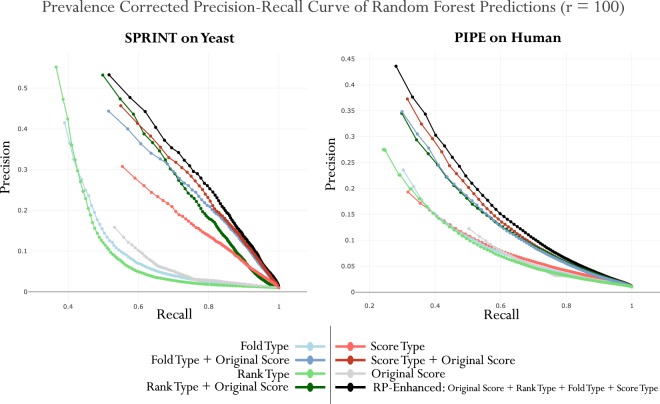
Precision-Recall Curves of Random Forest Results. Subsets of features were used to produce each curve, highlighting their contribution to the complete feature set.

To evaluate the improvement of the post-processing RP layer, we examine the contribution of each feature type and summarize the classification performance enhancement when utilizing RP-Enhanced context-based features. Figure 3 depicts the PRC curves of the cascaded Random Forest predictions results applying SPRINT to yeast and PIPE to human. We depict the predictions with curves illustrating the independent contribution of each feature type subset from Table 1. As can be clearly seen in these figures, the joint use of the original score and context, denoted RP-Enhanced, provides a consistent boost in recall over a wide range of precision values. We note that Rank and Fold types alone perform worse than the Original score, whereas Score type features perform better than the Original for SPRINT on Yeast. The Rank and Fold types only become useful when combined with the Original score. Finally, the RP-Enhanced combination of the Original score with all three RP metric types leads to the best overall performance. While each feature subset combined with the Original score exhibits a considerable increase in performance, it is the joint use of the Original prediction score in combination with all three proposed RP context features which substantially improves the classification performance.

To test for statistical significance of the difference between the non-RP and RP-Enhanced classification performance bootstrap testing was performed. A distribution of PRC-AUC and ROC-AUC summary statistics were formed by repeatedly (*n* = 1,000) sampling random subsets of PPIs, performing the cross-validation training and testing, and evaluating the resulting classification performance. Considering the null hypothesis, (*H*_0_: no significant difference in summary statistics between non-RP and RP-Enhanced) we computed *p*-values using Welch’s unequal variances t-test (to account for unequal variance in the resulting distributions) and found the observed differences in AUC to be significant at the *p* < 0.001 level, for all cases. These results are summarized in Table 3. Further improvement in predictive performance can be expected by individually tuning the Random Forest model for each dataset. Preliminary evidence for this is summarized in Supplementary Figure S1 (available online) which explores the feature landscape with respect to the Random Forest tree number parameter, *t*. In combination, these findings support the claim that the incorporation of context to derive post-processed features from the comprehensive predicted output can help further improve the classification results and these improvements promise to apply to any PPI predictor capable of generating these compressive predictions.

**Table 3:** Summary of PRC-AUC and ROC-AUC (*μ* ± *SE*) following 1,000 Bootstrap Iterations for each Method, Organism, and Feature Set.

Method	Organism	Features	PRC-AUC	ROC-AUC
PIPE	*H. Sapiens*	Original	0.3915 ± 0.0002	0.8737 ± 0.0001
		RP-Enhanced	0.4779 ± 0.0005	0.9510 ± 0.0001
	*S. cerevisiae*	Original	0.3155 ± 0.0001	0.8442 ± 0.0001
		RP-Enhanced	0.3358 ± 0.0001	0.9044 ± 0.0001
	*A. thaliana*	Original	0.2475 ± 0.0005	0.8351 ± 0.0004
		RP-Enhanced	0.5169 ± 0.0087	0.9815 ± 0.0001
	*C. elegans*	Original	0.3141 ± 0.0005	0.8685 ± 0.0003
		RP-Enhanced	0.4250 ± 0.0024	0.9400 ± 0.0002
	*M. musculus*	Original	0.2871 ± 0.0006	0.8386 ± 0.0005
		RP-Enhanced	0.4974 ± 0.0038	0.9806 ± 0.0001
SPRINT	*H. Sapiens*	Original	0.3432 ± 0.0001	0.8375 ± 0.0001
		RP-Enhanced	0.5001 ± 0.0005	0.9653 ± 0.0001
	*S. cerevisiae*	Original	0.2637 ± 0.0001	0.7732 ± 0.0001
		RP-Enhanced	0.2935 ± 0.0001	0.8995 ± 0.0001
	*A. thaliana*	Original	0.2367 ± 0.0004	0.8204 ± 0.0004
		RP-Enhanced	0.4430 ± 0.0088	0.9822 ± 0.0001
	*C. elegans*	Original	0.3101 ± 0.0004	0.8680 ± 0.0003
		RP-Enhanced	0.3700 ± 0.0023	0.9267 ± 0.0002
	*M. musculus*	Original	0.2820 ± 0.0005	0.8346 ± 0.0004
		RP-Enhanced	0.4909 ± 0.0053	0.9826 ± 0.0001

### Reciprocal Perspective Properties

The RP framework has been defined in such a way that it is amenable to any weighted complete graph problem. PPI predictions are an example of an undirected graph problem, however, the framework is extensible to directed graph problems. A directed graph problem can easily be converted into an undirected graph problem by averaging the values of the directed edges (*e.g*. score *A* ↔ *B* is the averaged value of the score for *A* → *B* and *B* → *A*). Due to the sensitivity of machine learning methods to the directionality of the inputs, we investigated the symmetry property of the RP method to account for the arbitrary selection of the paired ordering (choosing A-B vs. B-A). To quantify the difference of swapping the directionality of the paired relationships we ran permutation tests (*n* = 1,000) comparing the two directionalities across the four conditions (both organisms and methods) computing the percent difference over the same summary statistics ROC-AUC and PRC-AUC, in addition to the F1 Measure, and Test Accuracy. Across all conditions and metrics, the largest percent difference observed was 0.55% with an average of 0.33%. From these experiments, we conclude that the magnitude of these differences are negligible. However to eliminate them in future applications of RP, we consider combining the complimentary metrics into a single averaged value thereby guaranteeing the symmetry of the method.

The *NaRRO*, *NoRRO*, and *ARRO* features defined here were all similarly distributed, though differed in scale as a result of their respective normalization factors. This work limited the scope to intra-species predictions for which the *NaRRO* metric is most appropriate since we do not compare values between organisms. We however, emphasize the utility of the *ARRO* and *NoRRO* metrics for inter-species predictions and the comparison of PPI predictions between organisms. Future work will investigate their individual contribution to these prediction tasks and to support interpretation of results by experimentalists looking to curate a set of putative PPIs for experimental validation.

### Interpretability of PPI Results by Experimentalists

The current RP framework is a data-driven approach to leverage the context provided by jointly considering facets of the pair-wise PPI relationships. Following the transition from static to interactive graphs to enable additional exploration of empirical data^44^, the production of fully interactive visual depictions of these PPI results, as illustrated in the one-to-all curves, promise to facilitate exploration and interpretation when communicating results with experimentalists. For example, demonstrating that two under-represented proteins find themselves to be the highest scoring complimentary proteins in their respective perspectives, despite falling below the global threshold, would provide increased confidence in further investigating their putative interaction. Similarly, should they both find themselves in their respective baselines this would reinforce the likelihood that they are non-interactors. Future work will look to develop a visualization framework incorporating RP to serve as an intuitive interface for experimentalist to explore prediction results.

### Future Work

Applicable to any weighted complete graph problem exhibiting class imbalance, the RP framework can be applied to fields other than PPI prediction. A large number of multi-class multi-label problems can be modeled as complete bipartite graph problems, such as protein function prediction or disease prediction from electronic health records in a patient population. In each, RP could be leveraged to augment the predicted outcomes. Since the paired comparison of elements is prevalent in a wide breadth of fields, including social network analysis^45^ and two-market structures in economics^46^, we anticipate the utility of the RP framework in numerous applications.

## Conclusion

In this work, we suggest revising the assumption that a single global threshold can be appropriately defined across the proteome due to the inherent diversity of protein interaction profiles. In leveraging visualization techniques, we propose that the recent development of PPI predictors capable of generating the comprehensive set of putative interactions has given rise to context, enabling us now to evaluate a putative PPI within the context of all possible predictions. Leveraging this context, we introduce a novel modeling framework called Reciprocal Perspective, which estimates a localized threshold on a per-protein basis using several rank order metrics. We demonstrate that it significantly improves classification performance (ROC-AUC, PRC-AUC; *p* < 0.001) using two state-of-the-art PPI predictors, PIPE and SPRINT, over five organisms: *H. sapiens*, *S. cerevisiae*, *A. thaliana*, *C. elegans*, and *M. musculus*.

## Electronic supplementary material

Supplementary Figure S1
