# Supplementary material for: Reciprocal Perspective for Improved Protein-Protein Interaction Prediction
Source: Sci Rep. 2018 Aug 3;8:11694. doi: 10.1038/s41598-018-30044-1 (PMC6076239; doi:10.1038/s41598-018-30044-1)
Supplement: Supplementary file 1 — Supplementary Figure S1 [file 41598_2018_30044_MOESM1_ESM.pdf]

Supplementary Figure

Reciprocal Perspective for Improved Protein-Protein Interaction Prediction

Authors: Kevin Dick & James R. Green

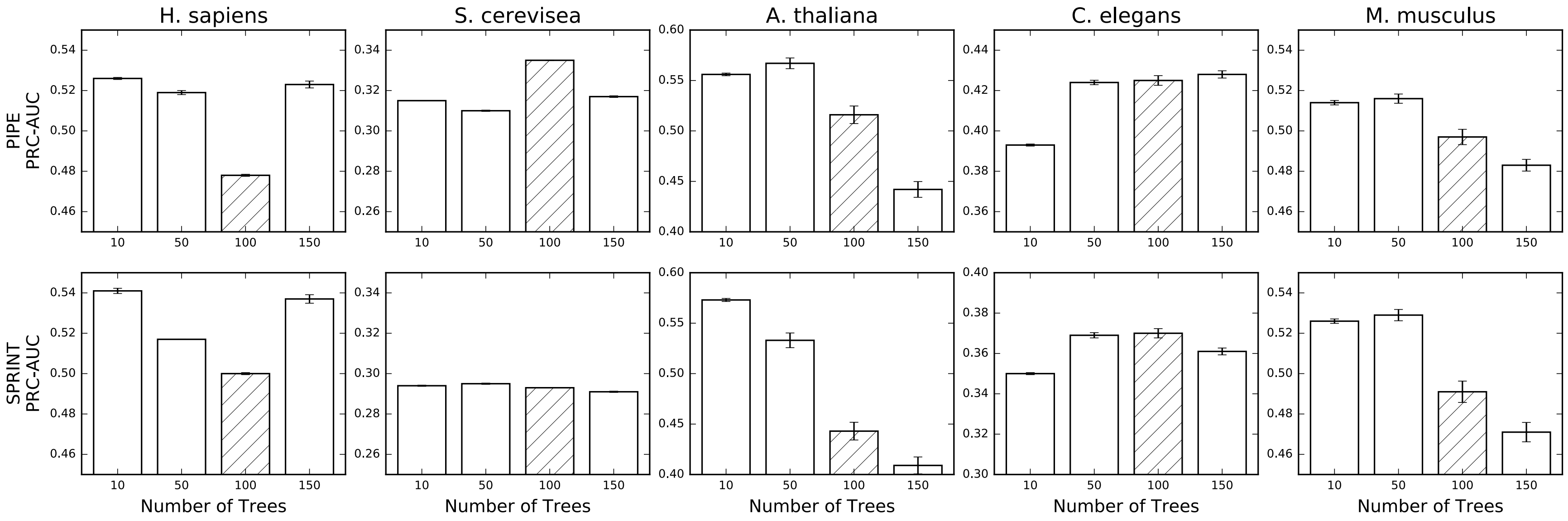

Supplementary Figure S1. Exploration of the Random Forest feature space with respect to tree number and PRC-AUC of the RP-Enhanced test condition ($\mu \pm SE$). All values obtained following 1,000 bootstrap iterations. The cross-hatched bars depict the selected value of 100 trees used to systematically compare all test conditions. Notably, no single tree number leads to the best performance across all ten datasets with a subset indicating an insensitivity to this hyperparameter.
